# Improving Two-Step Prepared CH_3_NH_3_PbI_3_ Perovskite Solar Cells by Co-Doping Potassium Halide and Water in PbI_2_ Layer

**DOI:** 10.3390/nano9050666

**Published:** 2019-04-27

**Authors:** Hsuan-Ta Wu, Yu-Ting Cheng, Ching-Chich Leu, Shih-Hsiung Wu, Chuan-Feng Shih

**Affiliations:** 1 Department of Electrical Engineering, National Cheng Kung University, Tainan 70101, Taiwan; n28004012@mail.ncku.edu.tw (H.-T.W.); n26041068@mail.ncku.edu.tw (Y.-T.C.); 2 Department of Chemical and Materials Engineering, National University of Kaohsiung, Kaohsiung 81148, Taiwan; 3 Green Energy and Environment Research Laboratories, Industrial Technology Research Institute, Hsinchu 31040, Taiwan; shihhsiung@itri.org.tw; 4 Hierarchical Green-Energy Materials (Hi-GEM) Research Center, National Cheng Kung University, Tainan 70101, Taiwan

**Keywords:** perovskite solar cells, water doping, potassium halide doping

## Abstract

Incorporating additives into organic halide perovskite solar cells is the typical approach to improve power conversion efficiency. In this paper, a methyl-ammonium lead iodide (CH_3_NH_3_PbI_3_, MAPbI_3_) organic perovskite film was fabricated using a two-step sequential process on top of the poly(3,4-ethylenedioxythiophene) polystyrene sulfonate (PEDOT:PSS) hole-transporting layer. Experimentally, water and potassium halides (KCl, KBr, and KI) were incorporated into the PbI_2_ precursor solution. With only 2 vol% water, the cell efficiency was effectively improved. Without water, the addition of all of the three potassium halides unanimously degraded the performance of the solar cells, although the crystallinity was improved. Co-doping with KI and water showed a pronounced improvement in crystallinity and the elimination of carrier traps, yielding a power conversion efficiency (PCE) of 13.9%, which was approximately 60% higher than the pristine reference cell. The effect of metal halide and water co-doping in the PbI_2_ layer on the performance of organic perovskite solar cells was studied. Raman and Fourier transform infrared spectroscopies indicated that a PbI_2_-dimethylformamide-water related adduct was formed upon co-doping. Photoluminescence enhancement was observed due to the co-doping of KI and water, indicating the defect density was reduced. Finally, the co-doping process was recommended for developing high-performance organic halide perovskite solar cells.

## 1. Introduction

The first report on lead halide organic perovskites for photovoltaic applications was published in 2009 [1]. Kojima et al. used methylammonium lead iodide (CH_3_NH_3_PbI_3_, MAPbI_3_) to replace organic dyes in dye-sensitized solar cells (DSSCs), where mesoporous titanium oxide (TiO_2_) and a liquid electrolyte were used, achieving a power conversion efficiency (PCE) of 3.8%. Recently, the best solar cell efficiency achieved was 22%, giving perovskites a reasonable chance to reach commercial competiveness [2]. PCE of the MAPbI_3_-based solar cell has been close to 20% in both mesoporous structure devices [3] as well as in planar heterojunction architectures [4]. High temperature annealing (>400 °C) is required to crystallize the TiO_2_ layers used in mesoporous-type solar cells. Compared to the high-temperature processing of mesoporous solar cells, the planar heterojunction perovskite photovoltaics has the advantage of a low-temperature (100 °C) solution process, and, therefore, can be adopted in the roll-to-roll production of flexible devices [5].

One key aspect that affects the performance of planar heterojunction perovskite solar cells is the quality of the organic perovskite film, which is determined by the thermodynamics and the growth kinetics of the film [6,7,8]. Solution-processed perovskite films usually have abundant defects when compared to single-crystal samples. The introduction of additives into the perovskite precursor solution was reported to be an effective way to prepare a high quality perovskite film with fewer defects, leading to enhanced device performance [4,9,10,11]. Potassium halides were wildly used as additives in the perovskite precursor solutions adopted for solar cell research [12,13,14]. Potassium halide-doped perovskite solar cells that have a record PCE of more than 20%, without I–V hysteresis, have been constructed [13,15]. Potassium halides were found to significantly facilitate the crystal growth of perovskite films and ameliorate the perovskite morphology, resulting in a reduced density in trap states and enhanced device performance [13,16]. However, it is not easy to form a homogeneous organic precursor by adding considerable amounts of potassium halide salts due to its restricted solubility in some organic solvent used for organic perovskite processing, which limits the application of potassium halides as additives. Water is a good solvent for potassium halide salts. Additionally, water additives have been reported to enhance the property of a two-step processed MAPbI_3_ [17,18]. Water additives changed the characteristics of dimethylformamide (DMF), which is a general solvent for PbI_2_, thus helping to make a homogeneous PbI_2_ solution. A smooth and dense PbI_2_ film was fabricated by adding a small amount of water into the PbI_2_ precursor with DMF, and then a high-quality MAPbI_3_ was obtained after the methylammonium iodide (MAI) conversion. Therefore, water was considered to be a suitable candidate for a co-doping additive for potassium halides during the MAPbI_3_ solution process.

However, it is challenging to deposit high-quality organic perovskite films on PEDOT:PSS, which is wildly used as the hole-transporting material in planar heterojunction perovskite solar cells. To obtain a homogeneous film structure on top of an organic surface, such as the PEDOT:PSS film, through a simple-solution process is not easy for an ionic material (such as MAPbI_3_) [17]. Compared to MAPbI_3_, PbI_2_ is less polar and, therefore, can easily form a continuous film on the PEDOT:PSS surface. Therefore, the two-step sequential process (PbI_2_ layer + MAI conversion) is an appropriate way to fabricate a perovskite film when PEDOT:PSS is used as the hole-transporting layer [17]. However, very few studies have reported the effects of water or potassium halide additives on two-step processed perovskite solar cells [19]. According to this report, the alkali metal halides (KCl, NaCl, and LiCl) were incorporated with the PbI_2_ layer and chelated with Pb^2+^ ions, enhancing the crystal growth of PbI_2_ films that, in turn, improved the crystallinity of the perovskite films and their photovoltaic properties. However, to the best of our knowledge, no report has investigated the performance of a planar heterojunction perovskite device by considering their interactive effects by using both water and potassium halides.

In this work, we proposed an effective way to enhance the efficiency of the MAPbI_3_-based perovskite device through the co-doping of water and potassium halides (KI, KBr, and KCl) during the PbI_2_ deposition process. Systematic studies of the effects of water and potassium halides co-doping on the thin film and device were investigated and discussed. To construct a planar heterojunction perovskite solar cell, a two-step process was employed to fabricate the MAPbI_3_ film. PbI_2_ was first deposited on PEDOT:PSS film, then the MAI was spin-coated on the PbI_2_ layer, followed by thermal annealing. Water and potassium halides were added into the PbI_2_ precursor solution to elucidate their influence on the performance of perovskite solar cells. As a result, the PCE of devices made from these additive-enhanced perovskites increased from 8.8% (based on pristine perovskite) to 13.9%.

## 2. Materials and Methods

### 2.1. Chemicals

Lead (II) iodide (PbI_2_, 99.9985%), potassium iodide (KI, 99.995%), and potassium chloride (KCl, 99.997%) were purchased from Alfa Aesar. Anhydrous N,N-dimethylformide (DMF, 99.8%), 2-propanol (IPA, 99.5%), and chlorobenzene (CB, 99.8%) were purchased from Sigma-Aldrich (Saint Louis, MO, USA). Potassium bromide (KBr, IR spectroscopic) was purchased from Honeywell (Morristown, NJ, USA). Ultra-pure wa ter (Resistivity > 18.2 MΩ·cm at 25 °C, Baker Analyzed LC/MS Reagent) was purchased from J.T. Baker (Radnor, PA, USA). Poly(3,4-ethylenedioxythiophene) polystyrene sulfonate (PEDOT:PSS), methylammonium iodide (MAI, >98%), bathocuproine (BCP, >99.5%), and phenyl-C61-butyric acid methyl ester (PC_61_BM, >99.5%) were purchased from Uni-onward (New Taipei City, Taiwan). All materials were used as received.

### 2.2. Device Fabrication

The fabrication process of the referenced standard perovskite solar cell (Ref) was as follows: patterned ITO-coated (indium tin oxide) glass substrates were cleaned ultrasonically in acetone and isopropanol for 10 min, and then dried in an oven for 15 min at 110 °C. After a UV-ozone treatment of 30 min, the substrates were transferred into a N_2_-filled glovebox. Filtered PEDOT:PSS was spin-coated at 2000 rpm onto the ITO substrate and baked on a hot plate at 110 °C for 10 min. The PbI_2_ precursor was prepared by dissolving PbI_2_ in the DMF solvent (370 mg/mL) and heated to 70 °C on a hot plate. Following this, the hot PbI_2_-DMF precursor was spin-coated on top of the PEDOT:PSS layer and baked on a hot plate at 90 °C for 10 min. The MAI (45 mg/mL in IPA) precursor was spin-coated on the crystalline PbI_2_ layer and annealed at 110 °C for 1 h to form the MAPbI_3_ perovskite. After annealing for 20 min, the pure IPA solution was spun on the MAPbI_3_ layer to wash out redundant MAI, and then the annealing process continued for 40 min. The PC_61_BM (20 mg/mL in CB) was spin-coated on the perovskite. BCP of 10 nm and Al of 150 nm were sequentially evaporated on the top with a deposition rate of 0.4 Å/s and 3–5 Å/s, respectively. For the co-doping samples, H_2_O with a 1, 2, or 3 volume percent, and potassium halide of various concentrations were added into the PbI_2_- DMF solution. The water added into the precursor was ultra-pure water. 

### 2.3. Characterizations

The crystallinity of the MAPbI_3_ film was examined by X-ray Diffraction (XRD) with ultraX 18 (Tokyo, Japan). The microstructure of the films was characterized by high resolution scanning electron microscope (HR-SEM) SU8000 (HITACHI, Tokyo, Japan). The electrical analysis was performed by E5270B Precision IV Analyzer (Keysight, Santa Rosa, CA, USA) under AM 1.5 G illumination. The absorption analysis was performed by UV-vis-NIR spectrophotometer U-4100 (HITACHI, Tokyo, Japan). The photoluminescence (PL) was performed by Jobin Yvon LabRAM HR micro-Raman system (HORIBA, Kyoto, Japan) and the excitation laser wavelength was set to 532 nm. The Fourier transform infrared spectroscopies (FTIRs) were performed by Nicolet FTIR instrument (Thermo Fisher Scientific, Waltham, MA, USA). The Raman spectroscopy was performed by Raman microscope (Renishaw, Wotton-under-Edge, UK). The depth profiles were performed by Auger Electron Spectroscopy MICROLAB 350 (Thermo Fisher Scientific, Waltham, MA, USA).

## 3. Results and Discussion

**Figure 1:**
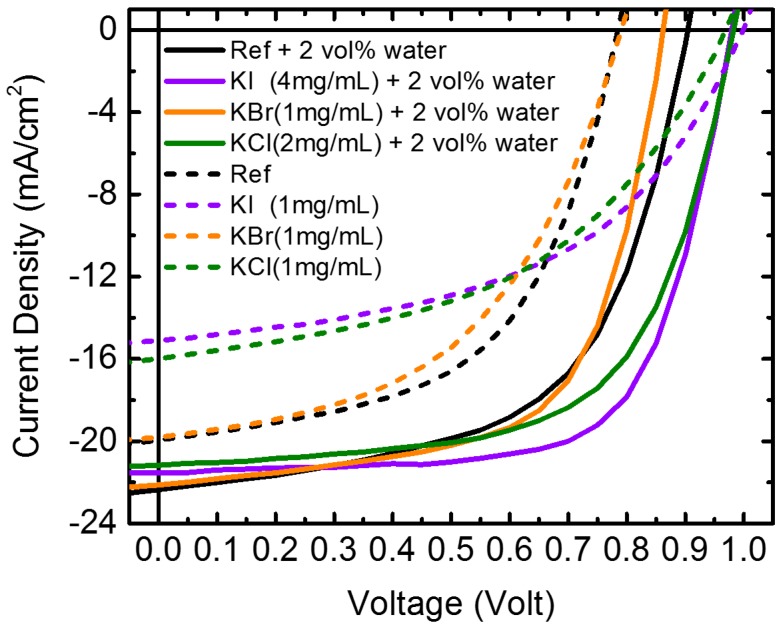
Photocurrent density–voltage (J–V) curves of perovskite solar cells prepared without and with water and/or potassium halide additives under their optimized concentrations.

The photovoltaic properties of the devices (structure: glass/ITO/PEDOT:PSS/MAPbI_3_/PC_61_BM/ BCP/Al) were investigated. Figure 1 displays the photocurrent density–voltage (J–V) curves of the perovskite solar cells prepared with and without the incorporation of water and potassium halide additives in the PbI_2_-DMF precursor. The concentration of each cell was at the optimal conditions for cell properties. Table 1 lists the corresponding cell parameters. The cell performance was measured using an aperture metal mask of 2.2 mm × 3.2 mm to a device active area of 2 mm × 3 mm. The PCE of device that was prepared using the 2 vol% water-doped PbI_2_ precursor increased from 8.8% (without water) to 12.0% with a short-circuit current density (J_SC_) of 22.5 mA·cm^−2^, an open-circuit voltage (V_OC_) of 0.9 V, and a fill factor (FF) of 59.2%. The photovoltaic performance, as a function of the water content, is illustrated in Figure S1, and the corresponding photovoltaic parameters are listed in Table S1. It was found that the PCE of devices that were prepared using a water content below 3 vol% were improved, and that the best performance was achieved by adding 2 vol% water in PbI_2_. The V_OC_ and FF increased slightly, and the J_SC_ increased markedly by increasing the water content to 2 vol%, resulting in the best PCE. Further increasing the water content to 3 vol% degraded the cell property. It has been reported that a planar heterojunction solar cell based on a high-quality perovskite film has a power conversion efficiency of 18% with a remarkably high FF value of 0.85 [17]. The study indicated that adding small amounts of water into the PbI_2_-DMF precursor made the solution more uniform, forming a smooth PbI_2_ film on top of the PEDOT:PSS, with high crystallinity and large crystalline domains. The perovskite film fabricated from the high-quality PbI_2_ film was highly pure and dense, without any pinhole. Our research showed similar observations. Water addition improved the morphology and crystallinity of the PbI_2_ films, as revealed in the SEM images (Figure S2). Moreover, the thickness of the PbI_2_ films increased with the content of water. The thickness was 156, 192, 189, and 194 nm for 0, 1, 2, and 3 vol% water incorporated PbI_2_ films, respectively. Figure S3 shows the MAPbI_3_ films fabricated from the water-doped PbI_2_ films. The grain size of all of the three water-incorporated films, Figure S3b–d, was larger than the film without water (Figure S3a). It has been observed that a rapid crystallization of organo-halide perovskites into the expected tetragonal cell for MAPbI_3_ occurred on exposure to small amounts of moisture [20]. You et al. have proposed a growth mode via thermal annealing of the perovskite precursor film in a humid environment (e.g., ambient air) to greatly improve the film quality, grain size, carrier mobility, and lifetime [21]. They indicated that, due to the strong hydroscopic nature of MAI, exposing the perovskite precursor to moisture during film formation could result in the accumulation of moisture within grain boundaries, inducing grain boundary creep and, subsequently, merging adjacent grains together. In addition, moisture could also provide an aqueous environment to enhance the diffusion length of the precursor ions, further promoting perovskite grain growth. The PbI_2_ films fabricated by the water-containing precursor possibly retained residual water or a water-related function group, which helped perovskite grain growth, until the deposition of MAI.

**Figure 2:**
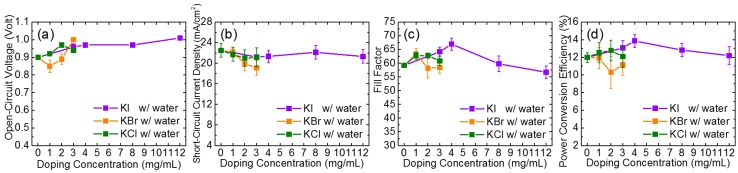
The changes in (**a**) *V*_OC_; (**b**) *J*_SC_; (**c**) FF (fill factor); (**d**) power conversion efficiency (PCE) of the potassium halide-doped devices with 2 vol% water co-doping as a function of doping amounts of KI, KBr, and KCl.

Conversely, the PCE of photovoltaic devices fabricated by the PbI_2_-DMF precursor with potassium halides (KI, KBr, and KCl) as the only additives became worse. As displayed in Figure 1, the PCE deteriorated from 8.8% for pristine MAPbI_3_ to 7.5%, 8.2%, and 8.0%, by adding 1 mg/mL KI, KBr, and KCl, respectively. For KCl- and KI-doped samples, the degradation of PCE was caused by the decrease in the J_SC_ and the fill factor, while for KBr-doped samples, only the fill factor decreased. Figure S4 illustrates the changes in photovoltaic performance of the potassium halide-doped devices as a function of the amount of potassium halide added. Notably, the devices were further improved by incorporating potassium halide into the water-PbI_2_-DMF precursor. By co-doping with 2 vol% water and potassium halides, the PCE was improved from 8.8% (pristine perovskite) to 13.9% (4 mg/mL KI), 11.9% (1 mg/mL KBr) and 12.8% (2 mg/mL KCl). The changes in the photovoltaic performance of the water and potassium halide co-doped devices as a function of the potassium halide additives is also illustrated in Figure 2. The performance of devices was enhanced by adding KI or KCl into the 2 vol% water-doped PbI_2_-DMF precursor, while KBr-doped devices had inferior properties than those without KBr. Using 4 mg/mL KI as the additive provided the best performance of the device. Figure S4 further plots the cell parameters as functions of the content of the potassium halides for devices prepared by PbI_2_ without water; all of the cell parameters degraded and Table S2 lists the corresponding photovoltaic parameters of the devices.

**Figure 3:**
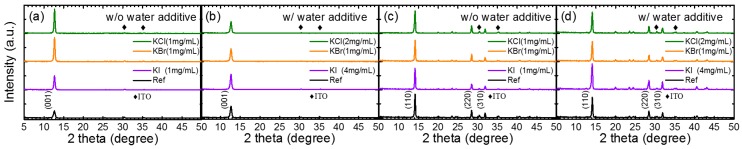
X-ray Diffraction (XRD) of PbI_2_ prepared by incorporating (**a**) KCl, KBr, and KI (without water) and (**b**) KCl, KBr, and KI with water additives. XRD of MAPbI_3_ on PbI_2_ prepared using the same conditions as (**a**) and (**b**) are shown in (**c**) and (**d**), respectively.

Figure 3 shows comprehensive XRD analyses of the potassium halides-doped PbI_2_ and MAPbI_3_ films with and without water incorporated with PbI_2_. The PbI_2_ and MAPbI_3_ films were deposited on ITO/PEDOT:PSS. Without water, the XRD intensity increased markedly for all the KCl-, KBr-, and KI-doped PbI_2_ films (Figure 3a). However, the XRD of the MAPbI_3_ films prepared on the potassium-doped PbI_2_ were similar to that without doping (Figure 3c). Water incorporation increased the XRD intensity of PbI_2,_ but did not affect the XRD intensity of the perovskite film (Figure 3a,c). Co-doping of water and potassium halide increased the XRD intensity of both the PbI_2_ and MAPbI_3_ significantly (Figure 3b,d). The increase in the XRD intensity was also accompanied by an increase in the grain size.

**Figure 4:**
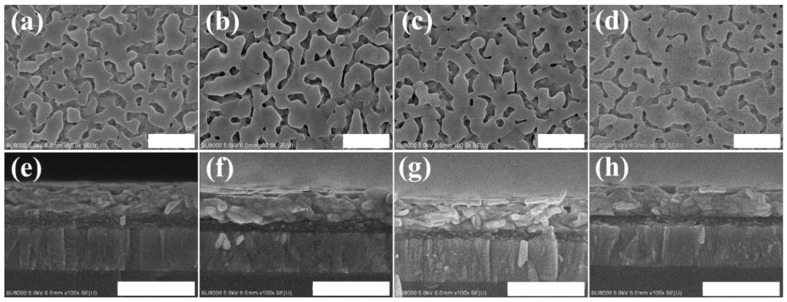
Plane and cross-section scanning electron microscope (SEM) images, respectively, of PbI_2_ prepared with (**a**,**e**) 2% water, (**b**,**f**) 2% water + KI (4 mg/mL) (**c**,**g**) 2% water + KBr (1 mg/mL), and (**d**,**h**) 2% water + KCl (2 mg/mL). (Scale bar = 500 nm).

**Figure 5:**
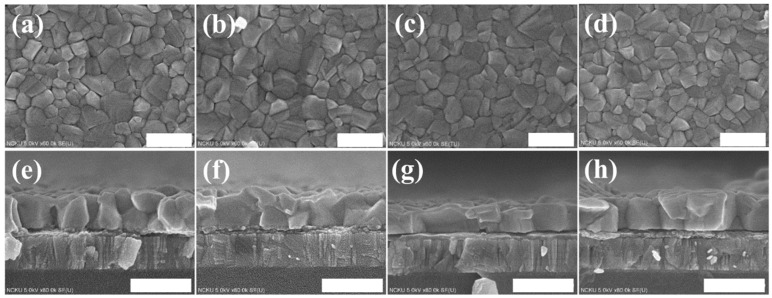
Plane and cross-section SEM images, respectively, of MAPbI_3_ on PbI_2_ with (**a**,**e**) 2% water, (**b**,**f**) 2% water + KI (4 mg/mL) (**c**,**g**) 2% water + KBr (1 mg/mL), and (**d**,**h**) 2% water + KCl (2 mg/mL). (Scale bar = 500 nm).

Figure 4 shows the top-view and cross-section SEM images of the PbI_2_ films that were co-doped with 2 vol% water and potassium halides (KI: 4 mg/mL, KBr: 1 mg/mL, and KCl: 2 mg/mL). The film thickness was unchanged by the potassium halides, but the surface coverage was improved and the grain size was enlarged, which agreed with the XRD observation. Figure 5 displays the SEM images of the co-doped films. Adding potassium halides (especially KI and KBr) to the 2 vol% water-incorporated PbI_2_ precursor enhanced MAPbI_3_ grain growth, which was denser with higher continuity, allowing for effective charge generation and dissociation in perovskite films. Such a highly continuous and large grain structure was also beneficial for carriers to transport through the film. 

**Figure 6:**
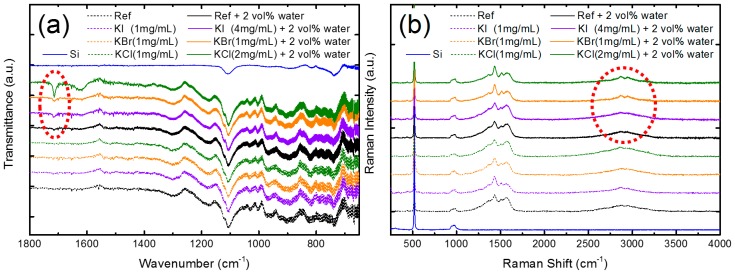
(**a**) Fourier transform infrared (FTIR) and (**b**) Raman spectroscopies of PbI_2_ films doped using potassium halides with and without water.

To exploit the effect of co-doping water and potassium halides in the PbI_2_ layer, the Fourier transform infrared spectroscopy and Raman spectroscopy were adopted, as shown in Figure 6. The PbI_2_ films were prepared on Si/PEDOT:PSS because glass absorbs the IR light. In Figure 6a, characteristic peaks related to the C=O stretching were found around 1715 cm^−1^ [22]. According to the literature, the C=O vibration shifts to lower frequency around 1650 cm^−1^ for DMF, which forms Lewis adducts due to the reaction of the PbI_2_ layer [23,24]. In our sample, the C=O stretching was found to be blue-shifted, which might be caused by the formation of a PbI_2_-DMF-H_2_O adduct by incorporating water through a Lewis acid–base reaction. The other possibility was that the PbI_2_-DMF precursor reacted with the PEDOT:PSS under-layer because of the incorporation of water by partially dissolving the PEDOT:PSS near the PbI_2_/PEDOT:PSS interface. A similar observation has been reported by Winther et al. [25]. Figure 6b shows the Raman spectra of the PbI_2_ layer with or without potassium halides and water. Si-related signals were found at 521 cm^−1^ (Si–Si LO), 940, and 987 cm^−1^ (Si–OH) [26]. Notably, the O–H and C–H stretching of PEDOT split into two peaks at 2865 and 2945 cm^−1^, owing to the incorporation of metal halides and water, particularly for KBr- and KCl-doping [27]. Evidence from FTIR and Raman spectroscopies indicated that the doping of water and potassium halides could form some new adducts and change the interfacial chemistry near the PbI_2_/PEDOT:PSS.

**Figure 7:**
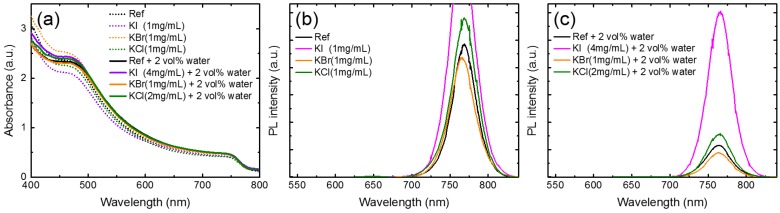
(**a**) Absorption spectra and (**b**,**c**) photoluminescence (PL) spectra of perovskite films prepared using PbI_2_ with different potassium halide and water additives. The samples were measured with this structure: glass/ITO/PEDOT:PSS/MAPbI_3_/PC_61_BM.

Figure 7 displays the absorption spectra (Figure 7a) and PL spectra (Figure 7b,c) of the perovskite-glass structures prepared on the PbI_2_ layers that were doped with water, potassium halides, and potassium halides and water co-dopants. In the visible region (470–800 nm), the absorbance of the MAPbI_3_ on PbI_2,_ prepared with the co-doped potassium halides and water, increased, indicating improved crystallinity. With potassium-only additives, the absorbance of MAPbI_3_ decreased. The PL-quenching effect of KBr-doping was observed (Figure 7b), while PL enhancement was found in the KI- and KCl-doped films. The steady-state photoluminescence (PL) was an effective way to detect the trap states within the perovskite layer. The higher PL intensity indicated fewer traps or defects within the films and improved crystallinity. Figure 7c shows the PL spectra of the co-doped water and potassium halides for MAPbI_3_ films. The PL intensity of the perovskite films obviously increased by co-doping, except for the KBr-doped film. In particular, the PL intensity of the co-doped KI and water increased by five times. Therefore, it was concluded that the co-doping of water and potassium halides increased the grain size of MAPbI_3_ and eliminated radiative defects that contributed to the strong PL response.

**Figure 8:**
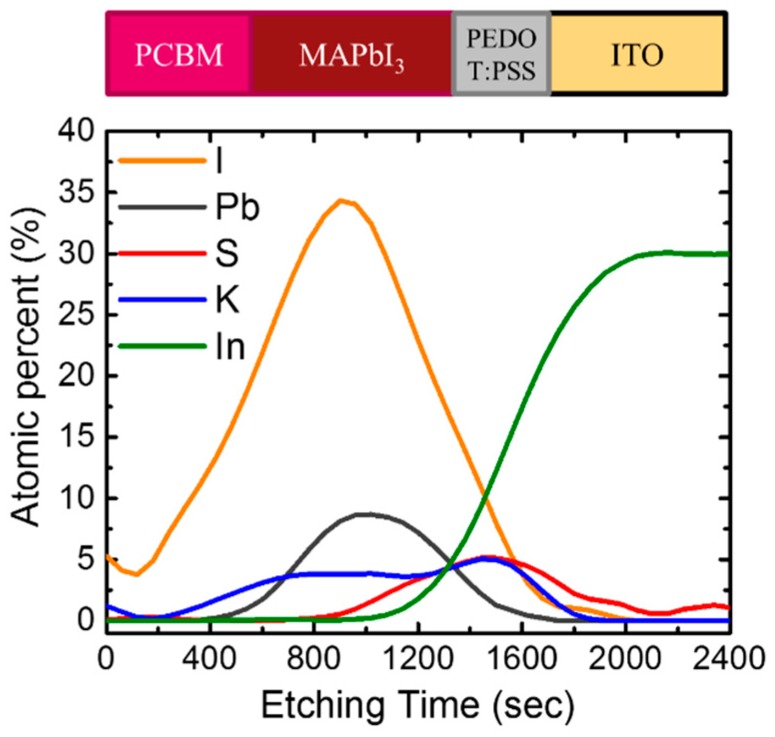
Auger electron spectroscopy (AES) depth profile of MAPbI_3_ prepared on PEDOT:PSS coated ITO (indium tin oxide) glass.

Auger electron spectroscopy (AES) was used to investigate the elementary depth distribution of the full device structure using KI and 2 vol% water co-doping, as shown in Figure 8. The potassium signal was found to be uniformly distributed across the perovskite and penetrated into the PEDOT:PSS. It has been reported that KI can provide an extra I-ion source that affects the coordination with Pb^2+^ and compensates for the I vacancy [15]. Therefore, in addition to the positive effect due to water- and potassium halides-induced grain growth, the enhancement of PL intensity can be associated with the passivation of the K^+^ cation and halide anions on the grain boundaries of perovskite film [15,16] and the perovskite interface, thus, reducing the trapped states. In other words, the addition of potassium halides and water improved the quality of the perovskite by reducing the traps and interfacial radiative recombination centers, allowing for effective charge generation and collection. Such a highly continuous and large grain structure was also beneficial for carriers to transport through the film. This improvement reduced series resistance (*R*_S_) and increased shunt resistance (*R*_SH_), improving the *J*_SC_, FF, and *V*_OC_ as observed in Table 1. Some studies partially attributed good photovoltaic performance to a significant absorption improvement because the use of additives led to a denser perovskite film with less pinholes [14,28]. However, our research results showed water and/or potassium halide additives only slightly enhanced the absorption of perovskite. The pristine MAPbI_3,_ prepared by our process, exhibited a compact microstructure with pure tetragonal structure phases, and the absorption effect was considered to be minor. It is worth noting that the KBr additive was harmful to the device, where even its physical properties, shown in Figure 3, Figure 4 and Figure 5, seemed as good as KI. We attributed this to the possible formation of a small amount of MAPbBr_3,_ through the incorporation of Br even though no secondary phase was found in the XRD spectra. The existence of MAPbBr_3_ within the MAPbI_3_ film may cause an energy barrier and inhibit charge transport from the perovskite layer to the ITO [12]. 

However, incorporation of potassium halides had little influence on the microstructure of the perovskite without any water additive. As shown in Figure S5, the surface morphology and the cross-section images of the films were similar, regardless of the types of potassium halides. The grain size was unchanged by the potassium halide additives, which is in agreement with the unchanged XRD FWHM (full width at half maximum) observation shown in Figure 3. The additive effect of each potassium halide can be clearly observed in Figures S6–S8. To conclude, the potassium halides had positive influences on the film quality and the device, only if they were co-doped with the water additive. Without water, the potassium halide additive made the solution more inhomogeneous, worsening the film properties. Because the solubility of potassium halides in PbI_2_-DMF was low, they were unevenly dispersed within the solution without water, forming an inhomogeneous film. The different optimal concentration of each potassium halide for the photovoltaic properties was influenced by their solubility.

## 4. Conclusions

In conclusion, a way to improve the perovskite solar cells (structure: glass/ITO/PEDOT:PSS/ MAPbI_3_/PC_61_BM/BCP/Al) was proposed by co-doping water and potassium halides in the PbI_2_ layer, which was coated on the PEDOT:PSS layer based in a two-step sequential process. When only potassium halides were added to PbI_2_, the PCE of the devices became worse, while the PCE of the devices prepared using the 2 vol% water-doped PbI_2_ precursor increased from 8.8% (without water) to 12.0%. By co-doping with the 2 vol% water and a potassium halide, the PCE was improved from 8.8% (pristine perovskite) to 13.9% (4 mg/mL KI), 11.9% (1 mg/mL KBr), and 12.8% (2 mg/mL KCl). XRD results showed that the incorporation of water and a potassium halide improved the crystallinity and enlarged the grain size. SEM images showed that the grain of PbI_2_ became coarse and continuous upon co-doping. FTIR spectra showed characteristic peaks, related to C=O stretching, around 1715 cm^−1^, which was probably caused by the formation of a PbI_2_–DMF–H_2_O adduct or an interfacial reaction near the PbI_2_/PEDOT:PSS interface. Raman spectra revealed that O–H and C–H stretching of PEDOT split into two peaks at 2865 and 2945 cm^−1^, owing to metal halides and water incorporation. Obvious PL enhancement was caused by the co-doping of water and KI, reducing the defect density. Together with the AES observations, it was found that KI distributed uniformly within the perovskite layer and penetrated into the PEDOT:PSS layer, which suggested that the elimination of the defects in the film and interface upon KI doping was one of the reasons to improve the KI–water co-doped solar cell. 

## Figures and Tables

**Table 1:** The corresponding photovoltaic parameters of devices in Figure 1.

Samples	*V*_OC_ (V)	*J*_SC_ (mA/cm^2^)	FF	PCE (%)	*R*_SH_ (Ω·cm^2^)	*R*_S_ (Ω·cm^2^)
Ref	0.79 ± 0.03	19.7 ± 1.4	56.3 ± 1.6	8.8 ± 1.1	340 ± 99	7.2 ± 0.7
KI (1 mg/mL)	0.99 ± 0.02	15.1 ± 0.8	50.1 ± 1.5	7.5 ± 0.6	262 ± 40	8.8 ± 1.6
KBr (1 mg/mL)	0.79 ± 0.05	19.8 ± 3.9	52.2 ± 4.8	8.2 ± 2.5	220 ± 70	11.7 ± 3.3
KCl (1 mg/mL)	0.96 ± 0.03	16.0 ± 1.0	52.4 ± 2.1	8.0 ± 0.6	337 ± 137	12.5 ± 1.8
Ref + water	0.90 ± 0.01	22.5 ± 1.3	59.2 ± 0.6	12.0 ± 0.6	168 ± 40	3.6 ± 0.1
KI (4 mg/mL) + water	0.97 ± 0.01	21.3 ± 1.2	67.0 ± 2.2	13.9 ± 0.7	555 ± 39	5.5 ± 0.7
KBr (1 mg/mL) + water	0.85 ± 0.04	22.1 ± 1.0	63.4 ± 1.9	11.9 ± 1.2	264 ± 46	3.8 ± 0.3
KCl (2 mg/mL) + water	0.97 ± 0.01	21.0 ± 1.6	62.8 ± 0.8	12.8 ± 1.2	1217 ± 106	4.7 ± 0.3

## Supplementary Materials

The following are available online at https://www.mdpi.com/2079-4991/9/5/666/s1: Figure S1: The tendency of photovoltaic performance of the water-doped devices as a function of water amount; Table S1: The corresponding photovoltaic parameters of devices in Figure S1; Figure S2: SEM morphology and cross-section of PbI_2_ films with (a,e) 0%, (b,f) 1%, (c,g) 2%, and (d,h) 3% water; Figure S3: SEM morphology of perovskite films prepared by PbI_2_ with (a) 0%, (b) 1%, (c) 2%, and (d) 3% water; Figure S4: The (a) *V*_OC_; (b) *J*_SC_; (c) FF; (d) PCE of the potassium halide-doped devices as a function of doping amounts of KI, KBr and KCl; Table S2: The corresponding photovoltaic parameters of devices in Figure S4 and Figure 2; Figure S5: The SEM cross-sectional images of the potassium halide-doped MAPbI_3_ films (without water additive), (a) ref, (b) 1 mg/mL KI, (c) 1 mg/mL KBr, and (d) 1 mg/mL KCl; Figure S6: SEM surface morphology of the perovskite films with different KI and water additives in the PbI_2_ layer; Figure S7: SEM surface morphology of the perovskite films with different KBr and water additives in the PbI_2_ layer; and Figure S8: SEM surface morphology of the perovskite films with different KCl and water additives in the PbI_2_ layer.
