# Supplementary material for: Improving Two-Step Prepared CH3NH3PbI3 Perovskite Solar Cells by Co-Doping Potassium Halide and Water in PbI2 Layer
Source: Nanomaterials (Basel). 2019 Apr 27;9(5):666. doi: 10.3390/nano9050666 (PMC6566305; doi:10.3390/nano9050666)
Supplement: Supplementary file 1 [file nanomaterials-09-00666-s001.pdf]

Supplementary Material

Improving Two-Step Prepared $CH_3NH_3PbI_3$ Perovskite Solar Cells by Co-Doping Potassium Halide and Water in PbI_2 Layer

Hsuan-Ta Wu [1], Yu-Ting Cheng [1], Ching-Chich Leu [2,*], Shih-Hsiung Wu [3] and Chuan-Feng Shih [1,4,*]

[1] Department of Electrical Engineering, National Cheng Kung University, Tainan, 70101, Taiwan; n28004012@mail.ncku.edu.tw (H.-T.W.); n26041068@mail.ncku.edu.tw (Y.-T.C.)
[2] Department of Chemical and Materials Engineering, National University of Kaohsiung, Kaohsiung, 81148, Taiwan
[3] Green Energy and Environment Research Laboratories, Industrial Technology Research Institute, Hsinchu, 31040, Taiwan; shihhsiung@itri.org.tw (S.-H.W.)
[4] Hierarchical Green-Energy Materials (Hi-GEM) Research Center, National Cheng Kung University, Tainan, 70101, Taiwan
* Correspondence: ccleu@nuk.edu.tw (C.-C.L.); cfshih@mail.ncku.edu.tw (C.-F.S.); Tel.: +886-7-5919456 (ext. 7456) (C.-C.L.); Tel.: +886-6-2757575 (ext. 62398) (C.-F.S.)

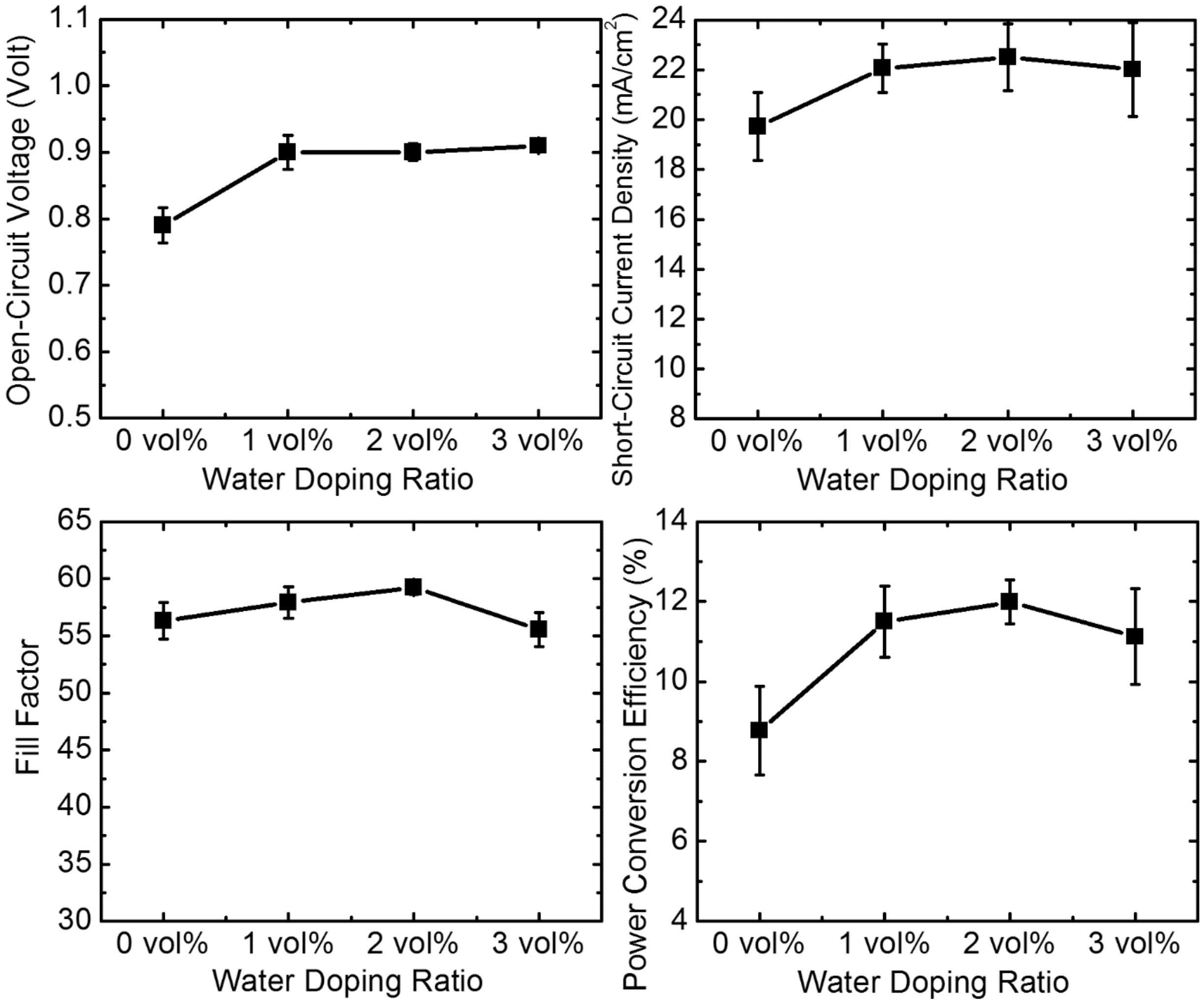

Figure S1. The tendency of photovoltaic performance of the water-doped devices as a function of water amount.

Table S1. The corresponding photovoltaic parameters of devices in Figure S1.

Samples	V_{OC} (Volt)	J_{SC} (mA/cm²)	FF	PCE (%)	R_{SH} (Ω·cm²)	R_S (Ω·cm²)
Ref	0.79 ± 0.03	19.7 ± 1.4	56.3 ± 1.6	8.8 ± 1.1	340 ± 99	7.2 ± 0.7
Ref + 1 vol% water	0.90 ± 0.03	22.1 ± 1.0	57.9 ± 1.4	11.5 ± 0.9	206 ± 53	4.6 ± 0.3
Ref + 2 vol% water	0.90 ± 0.01	22.5 ± 1.3	59.2 ± 0.6	12.0 ± 0.6	168 ± 40	3.6 ± 0.1
Ref + 3 vol% water	0.91 ± 0.01	22.0 ± 1.9	55.6 ± 1.5	11.2 ± 1.2	192 ± 55	4.9 ± 0.8

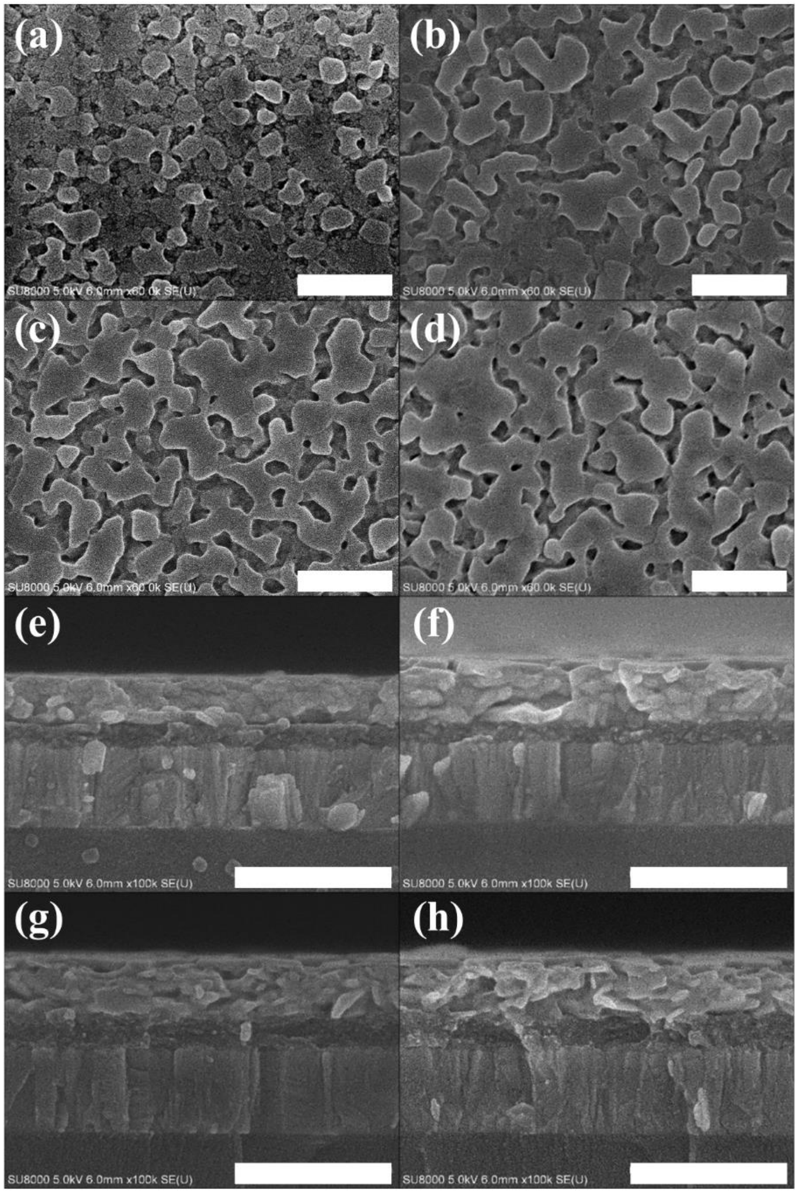

Figure S2. SEM morphology and cross-section of PbI_2 films with (**a**,**e**) 0%, (**b**,**f**) 1%, (**c**,**g**) 2% and (**d**,**h**) 3% water (scale bar = 500 nm).

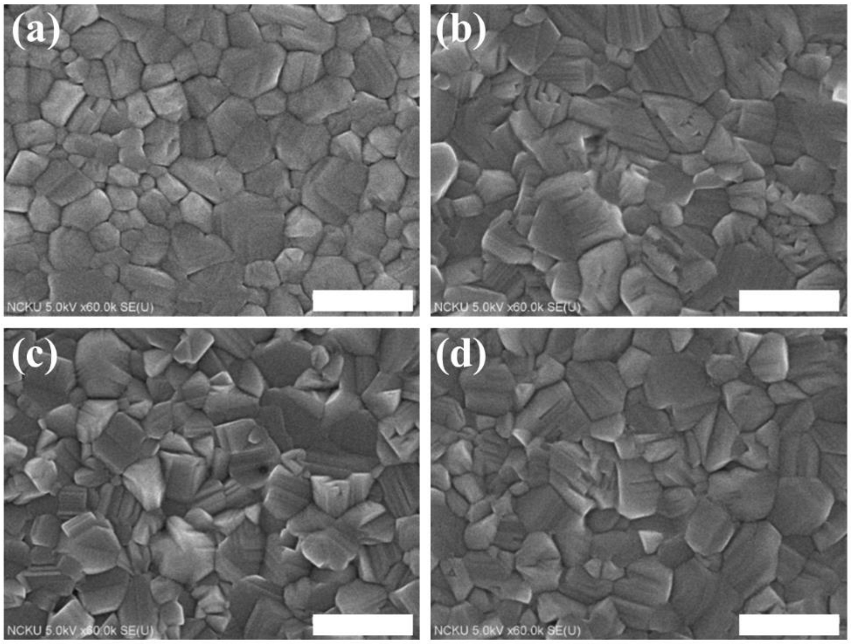

Figure S3. SEM morphology of perovskite films prepared by PbI_2 with (**a**) 0%, (**b**) 1%, (**c**) 2% and (**d**) 3% water (scale bar = 500 nm).

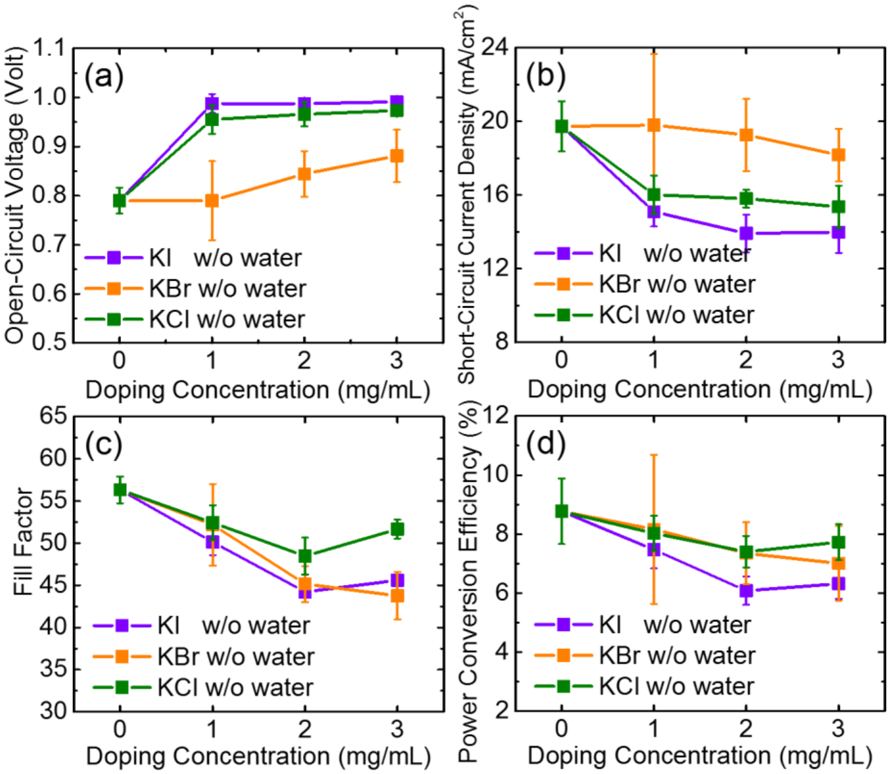

Figure S4. The (a) V_{OC}; (b) J_{SC}; (c) FF; (d) PCE of the potassium halide-doped devices as a function of doping amounts of KI, KBr and KCl.

Table S2. The corresponding photovoltaic parameters of devices in Figure S4 and Figure 2.

Samples	V_{OC} (Volt)	J_{SC} (mA/cm^2)	FF	PCE (%)	R_{SH} ($\Omega \cdot cm^2$)	R_S ($\Omega \cdot cm^2$)
Ref	0.79 ± 0.027	19.7 ± 1.4	56.3 ± 1.6	8.8 ± 1.1	340 ± 99	7.2 ± 0.7
KI (1 mg/mL)	0.99 ± 0.019	15.1 ± 0.8	50.1 ± 1.5	7.5 ± 0.6	262 ± 40	8.8 ± 1.6
KI (2 mg/mL)	0.99 ± 0.004	13.9 ± 1.0	44.2 ± 0.6	6.1 ± 0.5	165 ± 30	13.5 ± 1.1
KI (3 mg/mL)	0.99 ± 0.010	14.0 ± 1.1	52.2 ± 4.8	6.3 ± 0.5	203 ± 30	13.2 ± 0.8
KBr (1 mg/mL)	0.79 ± 0.080	19.8 ± 3.9	52.2 ± 4.8	8.2 ± 2.5	220 ± 70	11.7 ± 3.3
KBr (2 mg/mL)	0.84 ± 0.046	19.3 ± 2.0	45.2 ± 2.1	7.3 ± 1.1	162 ± 28	13.7 ± 3.7
KBr (3 mg/mL)	0.88 ± 0.053	18.2 ± 1.4	43.8 ± 2.8	7.0 ± 1.3	217 ± 45	15.2 ± 2.1
KCl (1 mg/mL)	0.96 ± 0.030	16.0 ± 1.0	52.4 ± 2.1	8.0 ± 0.6	337 ± 137	12.5 ± 1.8
KCl (2 mg/mL)	0.97 ± 0.024	15.8 ± 0.5	48.5 ± 2.2	7.4 ± 0.5	269 ± 86	10.4 ± 2.1
KCl 3 mg/mL)	0.97 ± 0.009	15.4 ± 1.2	51.7 ± 1.1	7.7 ± 0.6	209 ± 37	12.7 ± 1.4
Ref + 2 vol% water	0.90 ± 0.013	22.5 ± 1.3	59.2 ± 0.6	12.0 ± 0.6	168 ± 40	3.6 ± 0.1
KI (4 mg/mL) + 2 vol% water	0.97 ± 0.008	21.3 ± 1.2	67.0 ± 2.2	13.9 ± 0.7	555 ± 39	5.5 ± 0.7
KI (8 mg/mL) + 2 vol% water	0.97 ± 0.014	22.1 ± 1.3	59.8 ± 2.9	12.8 ± 0.7	878 ± 337	6.6 ± 0.6
KI (12 mg/mL) + 2 vol% water	1.01 ± 0.008	21.3 ± 1.3	56.7 ± 2.3	12.2 ± 1.0	498 ± 176	7.8 ± 1.2
KBr (1 mg/mL) + 2 vol% water	0.85 ± 0.035	22.1 ± 1.0	63.4 ± 1.9	11.9 ± 1.2	264 ± 46	3.8 ± 0.3
KBr (2 mg/mL) + 2 vol% water	0.89 ± 0.029	19.9 ± 1.4	58.2 ± 3.6	10.3 ± 1.8	310 ± 70	5.1 ± 0.6
KBr (3 mg/mL) + 2 vol% water	1.00 ± 0.006	19.1 ± 1.4	58.4 ± 2.1	11.2 ± 1.2	405 ± 66	5.9 ± 0.7
KCl (1 mg/mL) + 2 vol% water	0.92 ± 0.010	21.7 ± 1.3	62.8 ± 1.3	12.5 ± 1.2	809 ± 485	4.4 ± 0.3
KCl (2 mg/mL) + 2 vol% water	0.97 ± 0.007	21.0 ± 1.6	62.8 ± 0.8	12.8 ± 1.2	1217 ± 106	4.7 ± 0.3
KCl (3 mg/mL) + 2 vol% water	0.94 ± 0.006	21.1 ± 1.8	60.8 ± 1.8	12.1 ± 1.3	551 ± 207	5.6 ± 0.7

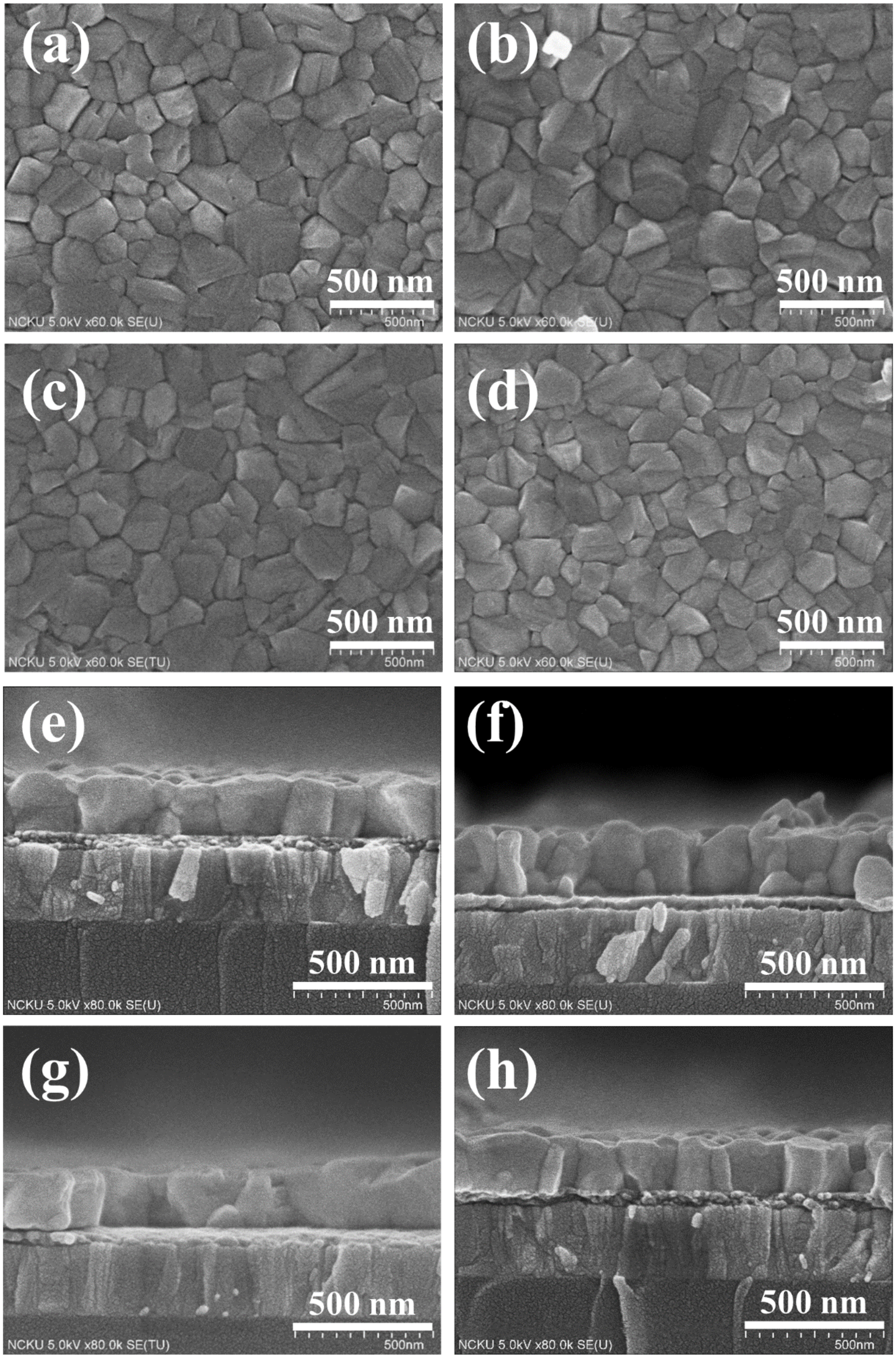

Figure S5. The SEM cross sectional images of the potassium halide-doped $MAPbI_3$ films (without water additive). (**a**) ref, (**b**) 1 mg/mL KI, (**c**) 1 mg/mL KBr, and (**d**) 1 mg/mL KCl.

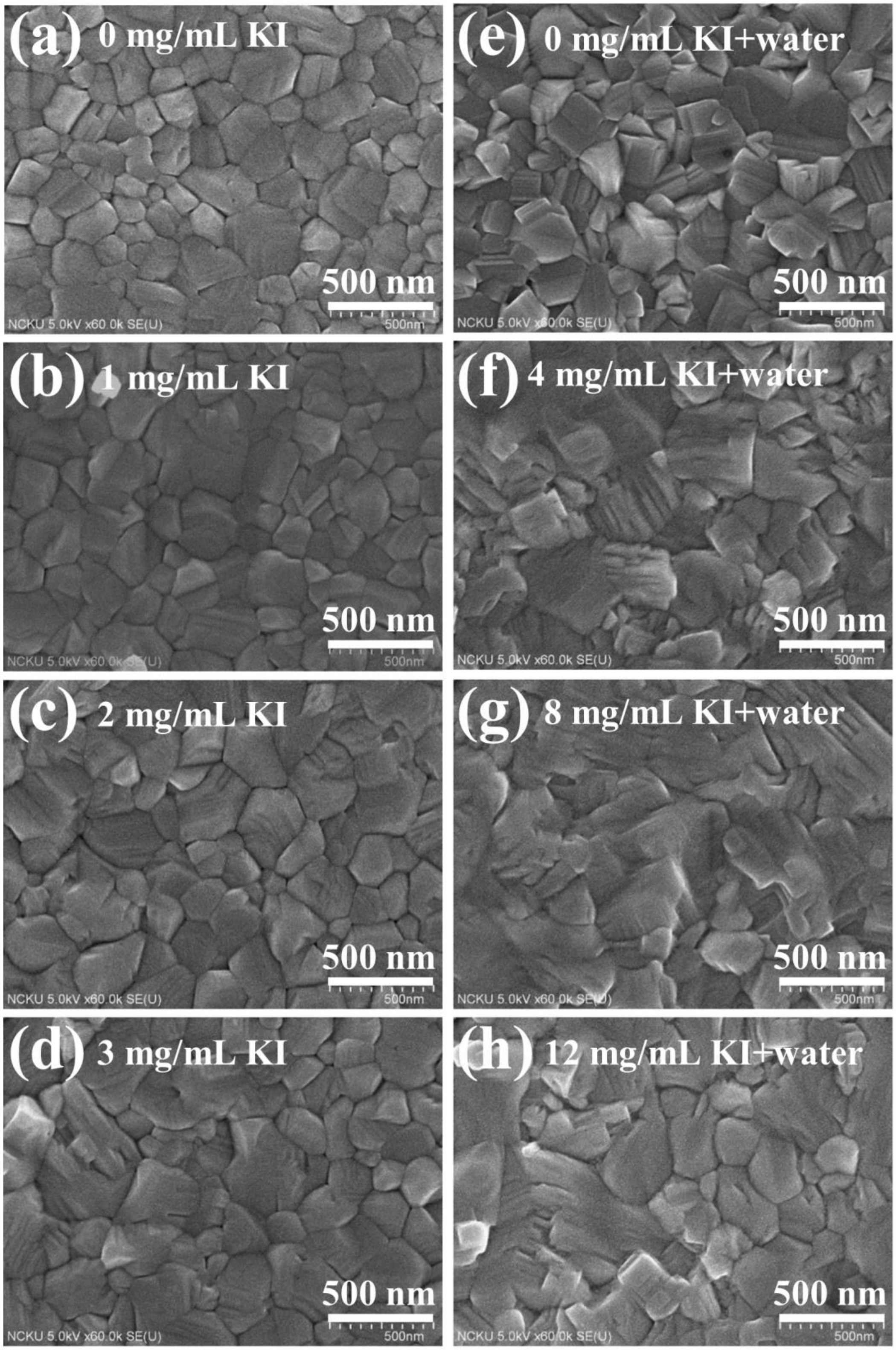

Figure S6. SEM surface morphology of the perovskite films with different KI and water additives in PbI_2 layer.

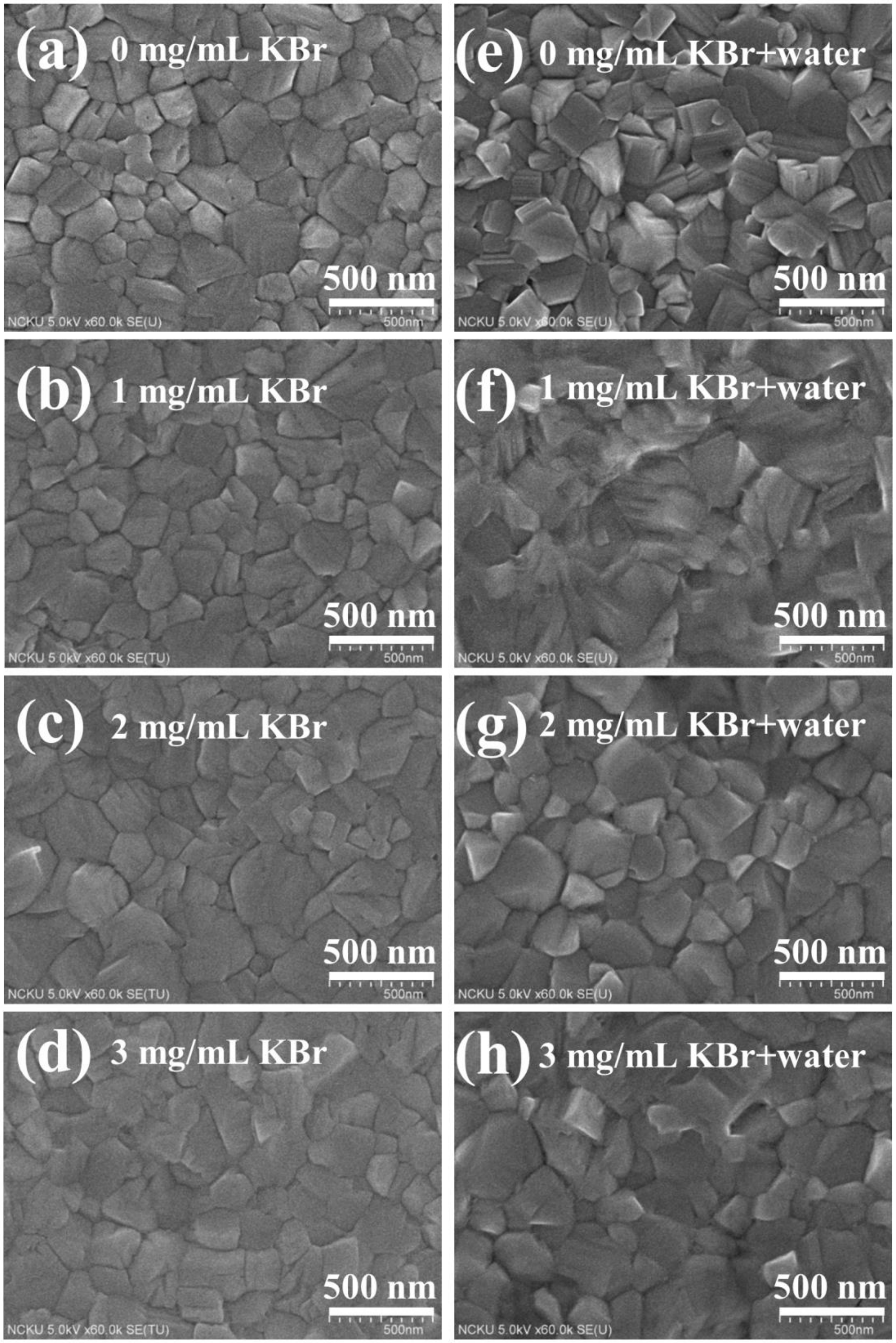

Figure S7. SEM surface morphology of the perovskite films with different KBr and water additives in PbI_2 layer.

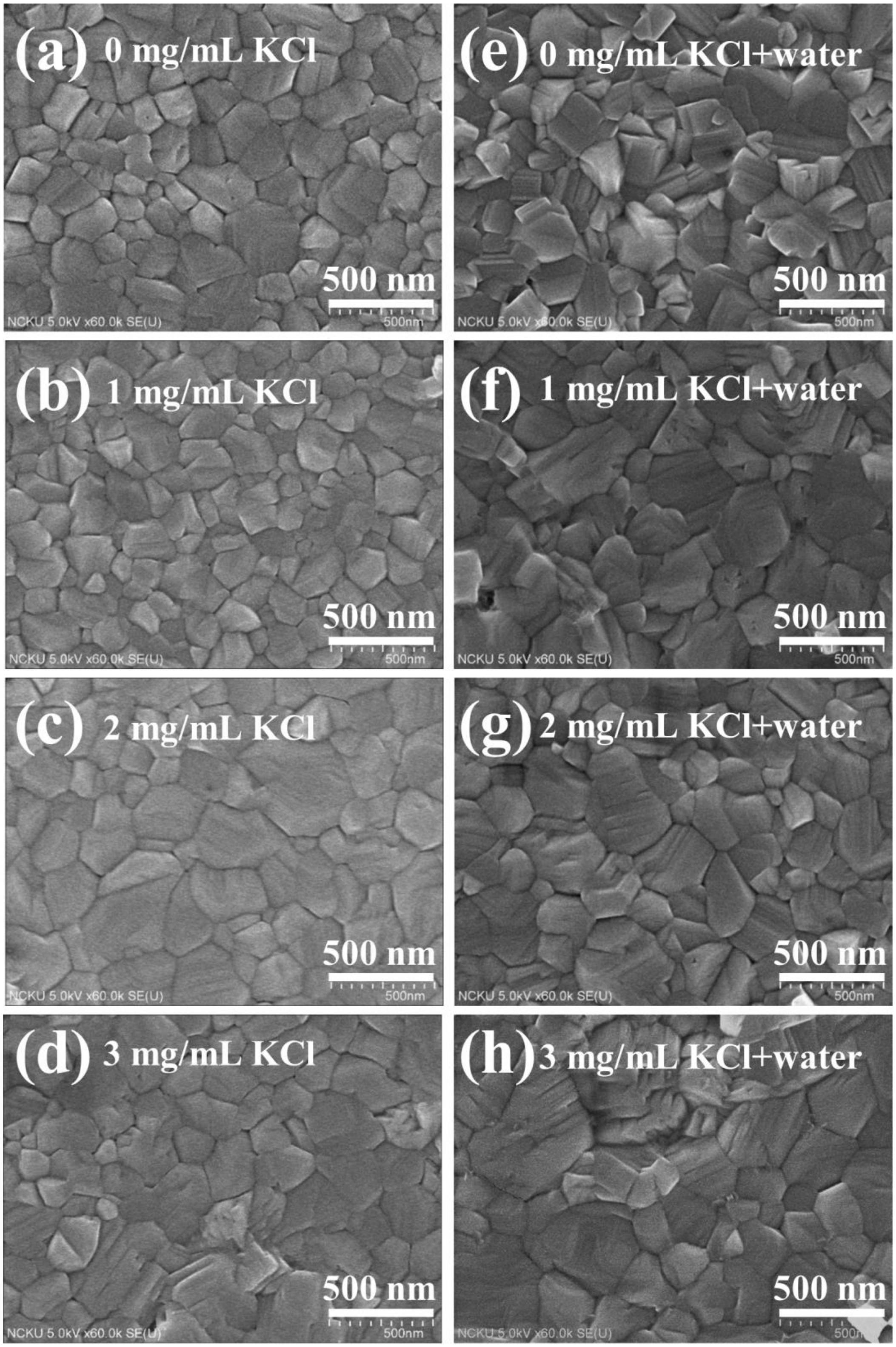

Figure S8. SEM surface morphology of the perovskite films with different KCl and water additives in PbI_2 layer.
